# A Model Predicting Mortality of Hospitalized Covid-19 Patients Four Days After Admission: Development, Internal and Temporal-External Validation

**DOI:** 10.3389/fcimb.2021.795026

**Published:** 2022-01-24

**Authors:** Stefan Heber, David Pereyra, Waltraud C. Schrottmaier, Kerstin Kammerer, Jonas Santol, Benedikt Rumpf, Erich Pawelka, Markus Hanna, Alexander Scholz, Markus Liu, Agnes Hell, Klara Heiplik, Benno Lickefett, Sebastian Havervall, Marianna T. Traugott, Matthias J. Neuböck, Christian Schörgenhofer, Tamara Seitz, Christa Firbas, Mario Karolyi, Günter Weiss, Bernd Jilma, Charlotte Thålin, Rosa Bellmann-Weiler, Helmut J. F. Salzer, Gero Szepannek, Michael J. M. Fischer, Alexander Zoufaly, Andreas Gleiss, Alice Assinger

**Affiliations:** ^1^ Institute of Physiology, Centre of Physiology and Pharmacology, Medical University of Vienna, Vienna, Austria; ^2^ Department of Vascular Biology and Thrombosis Research, Centre of Physiology and Pharmacology, Medical University of Vienna, Vienna, Austria; ^3^ Department of General Surgery, Division of Visceral Surgery, Medical University of Vienna, General Hospital Vienna, Vienna, Austria; ^4^ Department of Medicine IV, Kaiser Franz Josef Hospital, Vienna, Austria; ^5^ Division of Internal Medicine, Department of Clinical Sciences, Karolinska Institutet, Danderyd Hospital, Stockholm, Sweden; ^6^ Department of Pulmonology, Kepler University Hospital and Johannes Kepler University, Linz, Austria; ^7^ Department of Clinical Pharmacology, Medical University of Vienna, General Hospital Vienna, Vienna, Austria; ^8^ Department of Internal Medicine II, Medical University of Innsbruck, Innsbruck, Austria; ^9^ Institute of Applied Computer Science, Stralsund University of Applied Sciences, Stralsund, Germany; ^10^ Section for Clinical Biometrics, Center for Medical Statistics, Informatics, and Intelligent Systems, Medical University of Vienna, Vienna, Austria

**Keywords:** COVID-19, survival, prediction model, blood parameter, logistic regression, hospitalized patients

## Abstract

**Objective:**

To develop and validate a prognostic model for in-hospital mortality after four days based on age, fever at admission and five haematological parameters routinely measured in hospitalized Covid-19 patients during the first four days after admission.

**Methods:**

Haematological parameters measured during the first 4 days after admission were subjected to a linear mixed model to obtain patient-specific intercepts and slopes for each parameter. A prediction model was built using logistic regression with variable selection and shrinkage factor estimation supported by bootstrapping. Model development was based on 481 survivors and 97 non-survivors, hospitalized before the occurrence of mutations. Internal validation was done by 10-fold cross-validation. The model was temporally-externally validated in 299 survivors and 42 non-survivors hospitalized when the Alpha variant (B.1.1.7) was prevalent.

**Results:**

The final model included age, fever on admission as well as the slope or intercept of lactate dehydrogenase, platelet count, C-reactive protein, and creatinine. Tenfold cross validation resulted in a mean area under the receiver operating characteristic curve (AUROC) of 0.92, a mean calibration slope of 1.0023 and a Brier score of 0.076. At temporal-external validation, application of the previously developed model showed an AUROC of 0.88, a calibration slope of 0.95 and a Brier score of 0.073. Regarding the relative importance of the variables, the (apparent) variation in mortality explained by the six variables deduced from the haematological parameters measured during the first four days is higher (explained variation 0.295) than that of age (0.210).

**Conclusions:**

The presented model requires only variables routinely acquired in hospitals, which allows immediate and wide-spread use as a decision support for earlier discharge of low-risk patients to reduce the burden on the health care system.

**Clinical Trial Registration:**

Austrian Coronavirus Adaptive Clinical Trial (ACOVACT); ClinicalTrials.gov, identifier NCT04351724.

## Introduction

### Background

The Covid-19 pandemic evokes a complex global public health crisis with clinical, organizational, and system-wide challenges. Although vaccinations ease the situation, a substantial proportion of the world’s population is still not immunized. Recurrent Covid-19 waves challenge health care systems. Although age is a relatively strong biomarker, additional information on disease progression and patient outcome would be beneficial given the intensive workload of health care providers worldwide.

Although a plethora of prognostic models for Covid-19 were quickly published at the beginning of the pandemic to support medical decision making at a time when they were urgently needed, a large consortium including clinical scientists, epidemiologists, biologists, and statisticians, concluded that ‘almost all published prediction models are poorly reported, and at high risk of bias such that their reported predictive performance is probably optimistic’ (Wynants et al., 2020). However, they identified one prognostic model (Knight et al., 2020) that should soon be validated. The authors further recommended building on previous literature and expert opinion to select predictors, rather than selecting predictors in a purely data driven way. Promising candidates include age, body temperature, sex, blood pressure, creatinine, basophils, neutrophils, lymphocytes, alanine transaminase, albumin, platelets, eosinophils, calcium, bilirubin, creatinine, CRP, and comorbidities, including hypertension, diabetes, cardiovascular disease, and respiratory disease.

Besides the critically ill patients who need to be treated in intensive care units, the multitude of patients being treated in general wards binds substantial resources. Nevertheless, many of these Covid-19 cases cannot be discharged since the critical phase of Covid-19 frequently starts around 7-10 days after onset of the initial symptoms. However, a large proportion of patients will ultimately not require hospital care. Accordingly, a tool predicting the likelihood of a severe or fatal disease could support the decision for an earlier discharge. While such a predictive tool should be available as early as possible for hospitalized patients, the sole use of data available at time of hospital admission might not suffice in order to allow for a robust predictive accuracy. Hence, the expense of a slightly later forecast date might be relevant to exploit changes in biomarkers that may contain prognostic information.

### Objectives

The objective of this study was to develop a prognostic model with predictors selected based on pathophysiological considerations and literature. The model should also include the time course of the variables within the first four days after admission and only be applicable thereafter.

## Methods

### Participants and Source of the Data

We conducted an observational cohort study to develop and validate a prognostic model to predict in-hospital mortality of patients with Covid-19. Only data collected in clinical routine were used and data of all consecutive patients were accessed. The model was developed and internally validated in a cohort of the Clinic Favoriten in Vienna, Austria, hospitalized between 7 January 2020 and 8 December 2020, well before the widespread dissemination of the new corona variants. The cohort consisted of 679 patients, of which 578 including 98 deaths were used as they had at least two blood samples and survived at least 4 days.

The model was temporarily and externally validated in a mixed cohort consisting of additional patients from the Clinic Favoriten (350 patients, 58 deaths) and in patients from the Department of Pulmonology, Kepler University Hospital, Linz, Austria (97 patients, 9 deaths). Of these 447 patients, 392 were included in the analysis due to the above-mentioned reasons. These patients were hospitalized between 24 December 2020 and 07 April 2021, when the B.1.1.7/Alpha variant was more prevalent. SARS-CoV-2 positivity was determined from nasopharyngeal or oropharyngeal swabs *via* real-time polymerase chain reaction (qPCR) according to the Charité protocol (Corman et al., 2020). All patients had available outcome data at time of analysis. Recovery of data at the Clinic Favoriten in Vienna is part of the ACOVACT study (ClinicalTrials.gov NCT04351724) approved by the local ethics committee (EK1315/2020). This study was further approved by the ethics committee of the Kepler University Clinics (1085/2020).

### Outcome

The predicted outcome is death from any cause during the hospital stay after day 4. There was no loss to follow up as patients were either discharged or died.

### Predictors

The aim was to build a prognostic model that has widespread applicability. Thus, only the following routinely measured variables were considered: Age, Fever (>38°C) on admission, platelet count (PLT), C-reactive protein (CRP), lactate dehydrogenase (LDH), Creatinine (CREA), Lymphocyte count (LYM). Selection of possibly useful predictors considered pathophysiological processes, the published literature (Gansevoort and Hilbrands, 2020; Jiang et al., 2020; Manson et al., 2020; Wang, 2020; Fouladseresht et al., 2021), and had a special focus on the reported Covid-19-associated coagulopathy. The graphical exploration suggested that although some potential predictors are not out of range to a relevant extent at the time of admission, their development is considerably different between survivors and non-survivors during hospital stay. Therefore, the time course of PLT, CRP, LDH, CREA and LYM was included in the prognostic model. Since blood samples were often taken only every two days from admission, and as data from two days seemed too short potentially prognostic changes over time, the pragmatic decision was made to use the data from day 0 to 4 (i.e., 5 calendar days) after admission for prognosis. As summary measures, slopes and intercept at day 2 for each of the five blood-based parameters were estimated by linear mixed models.

### Blinding

The individuals accessing the medical records to extract variables were not blinded to the outcome.

### Sample Size

Sample size calculation was performed according to Riley et al. (2020). It was based on an anticipated proportion of deaths of 0.15, a desired margin of error in the overall outcome proportion estimate of 0.05, a mean absolute prediction error of 0.05, a shrinkage of 0.9, a Cox-Snell R squared statistic of 0.2 as anticipated model performance (maximum possible value of Cox-Snell R squared = 0.57), an expected optimism of 0.05 and 12 candidate predictors, i.e. age, fever on admission as well as intercept and slope for each of the blood based parameters. These assumptions resulted in a total sample size of 478 for model development.

### Missing Data

There were no missing data regarding outcome. For 101 of 679 patients only a single measurement of a blood parameter within days 0 to 4 was available, and these had to be excluded as no slope could be calculated. The model is thus only applicable to patients with at least two measurements within days 0 to 4.

### Statistical Analysis Methods

#### Estimation of Intercepts and Slopes by Linear Mixed Models

The predictors containing the information regarding the intra-individual level and change in the laboratory variables during the first 4 days after admission were calculated using a linear mixed model for each laboratory parameter as follows: First, time was re-scaled to zero at day 2 such that an estimated intercept represents a value in the middle (instead of the margin) of the interval between day 0 and day 4. Then each parameter was regressed on re-scaled time, using a fixed as well as a random intercept and slope with unstructured variance-covariance matrix. For these models, only patients with at least two observations up to day 4 were included. The mixed model approach deals with missing data due to varying measurement patterns (such that “missingness at random” is plausible) and shrinks slope estimates to the common mean obtained for the fixed effects. For each patient the intercept and slope were estimated as best linear unbiased predictors (BLUP) by the Empirical Bayes method (Fitzmaurice et al., 2011).

For future patients as well as for patients from a test set or validation cohort, intercept and slope were estimated using the empirical BLUP formula restricted to the available measurements after plugging in the parameter estimates obtained from the original data or training set (Fitzmaurice et al., 2011).

#### Building of Logistic Regression Based Prediction Model

**Figure 1:**
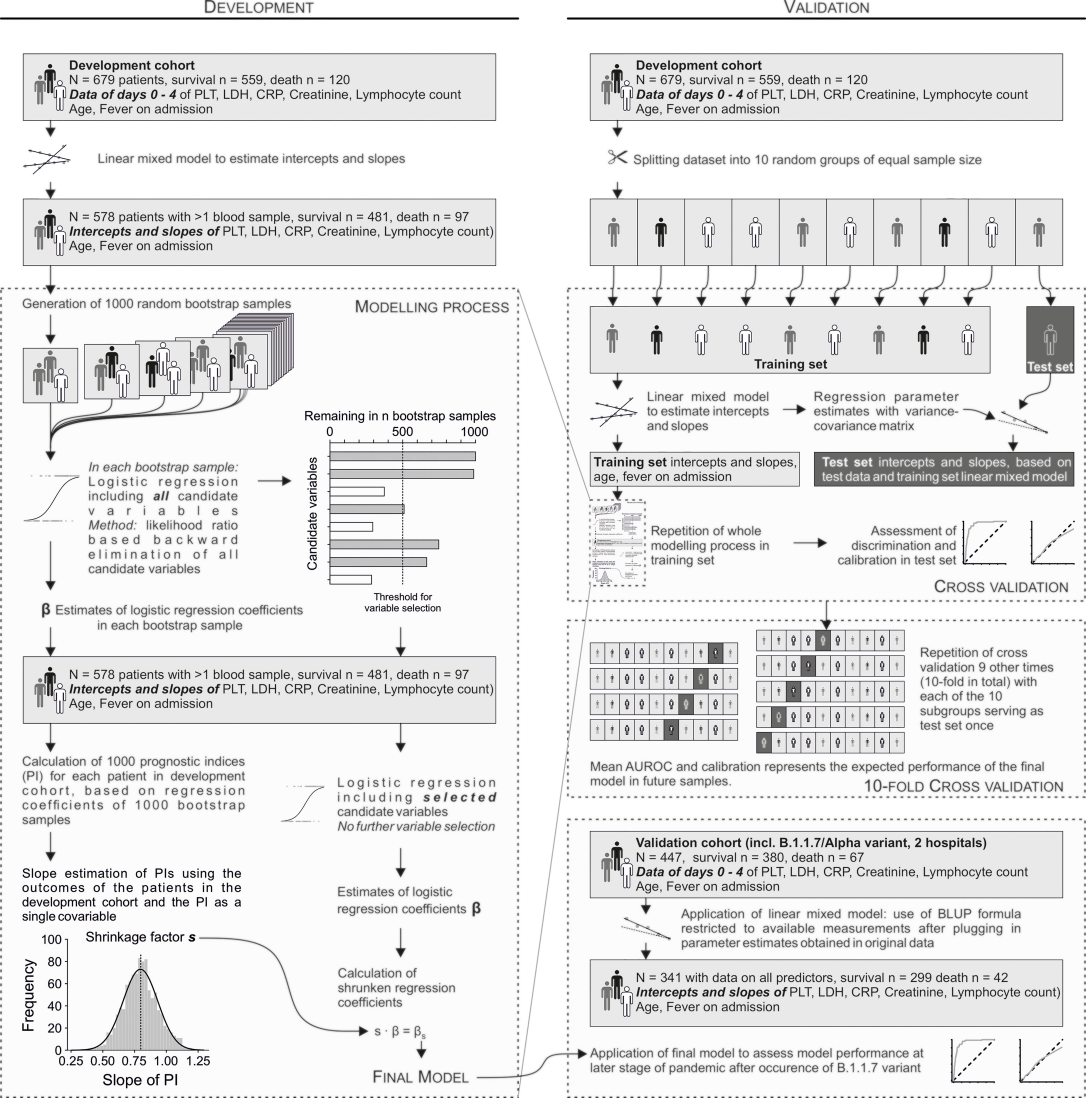
Model development and validation strategy.

The statistical approach is summarized in **Figure 1**. The first step of model development (**Figure 1** left) was to estimate intercepts and slopes for hematological parameters using linear mixed models as described below. Thereafter 1000 random bootstrap samples were generated. In each bootstrap sample, a logistic regression model was used to select predictors using backward elimination based on repeated likelihood ratio tests at a significance level of 0.1. For a predictor to be ultimately selected, it had to remain in at least 50% of all bootstrap samples. Hence, the final model was fit using the original dataset and the selected predictors only. A linear shrinkage factor for the regression coefficients was estimated as follows. Using the regression coefficients estimated in each of the 1000 bootstrap samples and the characteristics of each patient in the original sample, 1000 bootstrapped prognostic indices were calculated for each patient. Corresponding to each bootstrap sample we fit a logistic model to the original data with the bootstrapped prognostic indices as the only covariable. The mean regression coefficient of that covariable over the 1000 bootstrap repetitions was used as the linear shrinkage factor ‘s’ (Steyerberg et al., 2001). The logistic regression coefficients of the final model were multiplied by s, and the intercept recalibrated.

Further, the apparent explained variation (EV) together with degrees of necessity (DN) and of sufficiency (DS) were computed (Gleiss and Schemper, 2019). These measures were derived on the original data leading to considerable over-optimism but are able to quantify the *relative* importance of predictors.

Validation (**Figure 1** right) was first performed internally as 10-fold cross validation. First, the dataset including the raw blood values was randomly split into 10 groups of equal size. Next, 9 of these were used as training set, and the remaining one as test set. Importantly, separate linear mixed models were fitted in each training set to avoid data leakage between these sets. Thereafter, the whole modelling process (i.e. including bootstrapping and shrinkage) was repeated in the training set as described above for the original data. The resulting model was then validated in the training set. This procedure was repeated with each of the 10 random groups serving as test set once. The mean of the 10 resulting AUROCs on the respective test sets estimates the expected AUROC in a new dataset from the same target population. In addition to 10-fold cross validation, the final model was tested in a new dataset from a later period. The Brier score was computed by calculating the mean of the squared differences between predicted death probabilities and outcome (with death = 1 and survival = 0). Thus, it can take on a value between 0 and 1, whereby 0 indicates perfect prediction.

#### Handling of Predictors

For all continuous potential predictors, a linear functional form was assumed. Body temperature was only available as dichotomous variable fever on admission, ‘Yes’ was coded as 1, ‘No’ as 0.

### Differences Between Development and Validation

The cohorts differ regarding the period in which patients were hospitalized and the virus variants.

### Machine Learning

In order to benchmark the model, in addition random forests (Liaw and Wiener, 2002) were trained as they achieve good performance in many machine learning benchmark studies (Fernández-Delgado et al., 2014). As random forests are less sensitive to hyperparameter tuning compared to other machine learning algorithms (Szepannek, 2017; Probst et al., 2019), forests were trained using the default parameterization $\left(ntree=500\text{ and }mtry-\sqrt{\#\;variables}\right)$ . Details on random forests can be found in the **Online Supplement**.

## Results

### Participants

An overview of the cohorts including the number of survivors and non-survivors is shown in **Figure 1**. Patient characteristics are listed in **Table 1**.

**Table 1:** Patient characteristics development cohort.

Parameter	Survivors (N=559)	Non-survivors (N=120)
	% / Median (lQR)	% / Median (IQR)
**Sex**		
Female	40	36
Male	60	64
**Age (years)**	58 (44-72)	81 (77-89)
**BMI**	27 (24-31)	26 (25-32)
**Comorbidities**		
Current smoker	8	10
Overweight (BMI > 25)	62	64
Diabetes type II	18	36
Hypertension	45	74
Coronary heart disease	12	30
Chronic heart failure	6	26
Atrial fibrillation	14	38
Peripheral arterial disease	5	14
Chronic obstructive pulmonary disease	11	14
Asthma	4	6
Hypo- / Hyperthyroidism	8	10
Chronic renal insufficiency	10	44
Chronic liver disease	4	6
Malignancy	9	18
**Symptoms at admission**		
Asymptomatic	12	2
Fatigue	57	67
Cough	61	51
Fever	52	60
Requirement of oxygen	35	70
Dyspnea	43	41
Diarrhea	16	6
Sore throat	14	0
Nausea or vomiting	7	0
**Predictors upon admission**		
Platelet count (10^3^/ µI)	195 (154-264)	178 (138-220)
CRP (mg/l)	49 (25-88)	60 (33-169)
Creatinine (mg/dl)	0.9 (0.8 - 1.1)	1.4 (1.1-1.8)
LDH (U/l)	283 (231-379)	326 (237-370)

### Model Development

**Figure 2:**
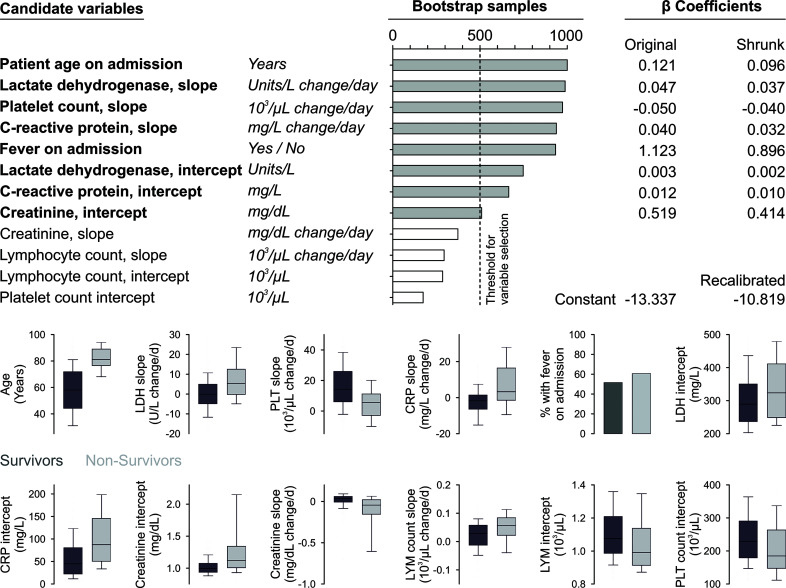
Model development. Candidate variables in bold were selected as they remained in the model after backward elimination in more than 50% of all bootstrap samples. Coefficients were shrunk according to a linear shrinkage factor, which was 0.7974 (Steyerberg et al., 2001). The constant was recalibrated that the mean estimated mortality probability corresponded to the mortality in the training sample. The boxplots show median, interquartile range as well as 10^th^ and 90^th^ percentile of continuous candidate variables or percentages for fever on admission according to outcome.

An exploratory analysis showed that platelet count increases over time in survivors compared to non-survivors, while survivors showed a decrease in CRP-levels and non-survivors an increase. This observation motivated us to investigate intercepts and slopes of variables as potential predictors of survival. Twelve subject matter-based predictors were pre-selected to be narrowed down by the described bootstrap approach. Sample size estimation showed that the number of events sufficed for this number of predictors. The predictors with a selection frequency higher than 50% were selected for the final model (**Figure 2**), the shrunk regression coefficients represent the final model.

The apparent variation in mortality explained by all variables selected for the final model amounts to 0.493. Age is the most important predictor with a marginal explained variation (EV) of 0.210 and a high degree of necessity (DN=0.734) and low sufficiency (DS=0.274). All six predictors derived from laboratory parameters together explain 0.295 of variation in mortality with moderately high necessity (DN=0.658) and moderate sufficiency (DS=0.395). Fever on admission is the least important predictor (EV=0.007).

### Validation

**Figure 3:**
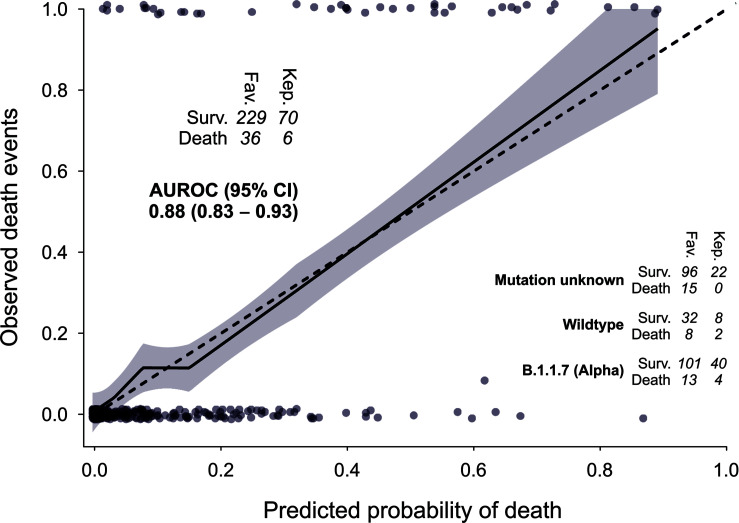
Model performance. Discrimination and calibration in another cohort admitted to hospital while the B.1.1.7/Alpha variant of SARS CoV-2 was widespread. Fav., Clinic Favoriten; Kep., Kepler University Clinics.

Tenfold cross validation resulted in a mean AUROC of 0.92, a mean calibration slope of 1.0023 and a mean Brier score of 0.076. In a subsequent cohort, partly from another hospital in Austria and from a period in which the B.1.1.7/Alpha variant of SARS CoV-2 was prevalent, application of the previously developed model showed an AUROC of 0.88, a calibration slope of 0.95 and a Brier score of 0.073 (**Figure 3** and **Table 2**).

**Table 2:** Predictors in temporal-external validation.

Predictor	Unit	Survivors (N=299)	Non-survivors (N=42)
		% / Median (IQR)	% / Median (IQR)
Patient age on admission	Years	59.00 (49.00 - 73.81)	80.44 (73.00 - 88.92)
Lactate dehydrogenase, slope	Units/L change/day	-1.27 (-9.69 - 4.04)	3.78 (-3.78 - 10.73)
Platelet count, slope	10^3^/μL change/day	16.21 (6.95 - 25.66)	11.88 (5.39 - 25.21)
C-reactive protein, slope	mg/L change/day	-2.66 (-10.46 - 0.31)	-7.16 (-14.04-0.36)
Fever on admission		63.5	52.4
Lactate dehydrogenase, intercept	Units/L	31 1.52 (253.39 - 400.49)	439.72 (326.59 - 513.26)
C-reactive protein, intercept	mg/L	32.86 (13.92 - 61.43)	68.19 (53.97 - 94.61)
Creatinine, intercept	mg/dL	0.85 (0.72 - 1.00)	1.02 (0.85 - 1.50)

### Benchmarking Using Machine Learning

Using random forests instead of logistic regression did not result in an increased performance in samples used for 10-fold cross validation. In the cohort used for temporal-external validation, random forests showed a nearly identical performance with strongly overlapping (bootstrap-)confidence intervals for the AUROC (0.88, 95% CI 0.83 - 0.93) with the proposed regression model. Furthermore, accumulated local effects plots (Apley and Zhu, 2020) from the forest confirmed the predictors’ effects of our model (**Online Supplement**).

## Discussion

Herein we present a calculator that predicts the risk of death of hospitalized patients with Covid-19 within the period of their stay. It uses the data of the day of admission and the four subsequent days and can therefore be used thereafter as additional decision support regarding discharge of clinically stable Covid-19 patients in case adequate therapy is also available at home. The formula is based on predictors routinely measured in hospitals or naturally available on admission, namely patient age on admission, lactate dehydrogenase, platelet count, C-reactive protein, presence of fever, and creatinine, which allows immediate and widespread use. This is also facilitated by a publicly available online calculator.

The selection of variables to be further narrowed down by bootstrapping was based on pathophysiological considerations. The underlying pathogenesis of Covid-19 seems complex, yet four main intertwined loops (the viral, the hyperinflammatory, the non-canonical renin-angiotensin system (RAS) axis and the hypercoagulatory loop) responsible for patient deterioration have been identified. Three out of the four loops are represented in the presented model. The pathology starts with the viral loop and is rapidly followed by the second loop, the hyperinflammatory loop, which is represented by CRP. Lymphocyte counts have been suggested previously as prognostic markers as well, constituting a major line of defense against viruses (Fouladseresht et al., 2021). Further, LDH is related to inflammation and cell damage and has been suggested as a risk factor for severe Covid-19 (Chen et al., 2020; Poggiali et al., 2020). In addition, the third loop, the non-canonical renin-angiotensin system (RAS) axis loop was described, which is in a broader sense represented by creatinine in our model. Kidney involvement in Covid-19 is common and associated with high mortality and was described to serve as an independent risk factor for all-cause in-hospital mortality in patients with Covid-19 (Ali et al., 2020). Renal viral tropism has been reported, which is also associated with age and comorbidities as well as decreased survival (Braun et al., 2020). Data of more than 17 million people in the UK suggest that patients with chronic kidney disease are at higher risk for adverse events in Covid-19 than those with other known risk factors, including chronic heart and lung disease (Gansevoort and Hilbrands, 2020). The fact that the slope of creatinine seemed to have less prognostic value than the intercept might reflect the importance of chronic kidney disease. The fourth loop is the hypercoagulatory loop, which is represented by platelet count in this model. A meta-analysis of 7,613 Covid-19 patients revealed that patients with severe disease had a lower platelet count than those with non-severe disease (Jiang et al., 2020), which is in line with our data. However, not all studies have found platelet counts to be a predictor of Covid-19 mortality (Amgalan and Othman, 2020). Undoubtedly the most important predictor of severe Covid-19 is age. A meta-analysis of 88 articles (69,762 patients) shows that age along with CRP were strong risk-factors for ICU admission and/or mortality (Katzenschlager et al., 2020). Concerning fever, a recent meta-analysis reported that fever is a predictor of adverse outcome in Covid-19 (Li et al., 2020). In line with studies on other viral infectious diseases, a study found that prolonged fever for 7 days from onset of illness is associated with adverse outcomes from Covid-19, while saddleback fever is not indicative of adverse outcome (Ng et al., 2020). In our model fever at admission was incorporated in the prediction model. The time course of body temperature would have been interesting to include, however, available records only allowed inclusion as a binary variable. As our data show, many differences between survivors and non-survivors only develop over the course of a few days, and Mueller et al. (2020) showed that trends in inflammatory biomarkers precede respiratory failure. Thus, we sought to include the time courses of variables as predictors.

Regarding the relative importance of the variables included in our final model, the (apparent) variation in mortality explained by the six variables deduced from the laboratory parameters measured during the first four days is slightly higher than that of age. While our data confirm that high age is a principal risk factor for dying from Covid-19, these laboratory variables are able to add considerably to the sufficiency of age and, thus, to the predictive importance of the model.

Concerning machine learning, the results of the benchmark do not show a performance increase by using random forests instead of logistic regression and thus confirm the conclusions from Bücker et al. (2021) to carefully analyze the benefits of using more complex models and to prefer simple models such as the shrunk logistic regression model otherwise.

### Limitations

Our prognostic model is far from being the first. Compared to other well developed and validated models, e.g., the 4C mortality Score (Knight et al., 2020), ours tends to distinguish patients who die from those who survive better than many others, indicated by an externally validated c index significantly above 0.8. However, this good performance may be geographically limited, for example due to differences in health care systems that lead to varying periods between infection and admission and consequently to different disease stages upon admission. As a result, we can only recommend the use of the model in Austria before the model has been validated in or updated for other regions. Of note, the calibration plot indicates slight under-estimation of death probabilities in the upper range of death probabilities.

A major aspect that discriminates our model from others is the use of the time course of biological parameters as predictors. Many others included the values at admission exclusively, which is reasonable, as information regarding prognosis should generally be available as early as possible. Thus, it might be viewed as a drawback of our model that decision making takes until day 4 of hospitalization. However, we think that the time course of variables after admission generally contains information regarding future disease progression. Considering that based on over 10,000 patients from Germany (Karagiannidis et al., 2020), even the less critical non-ventilated patients have a median stay of 9 days, it seems reasonable to improve prognostic accuracy by delaying the prognosis time point by 4 days.

Further, it remains uncertain whether further mutations of SARS CoV-2 might render the model unsuitable. However, this is a general problem regarding prediction models for rapidly developing diseases, and it may require frequent recalibration of models. The currently dominating Delta-variant is not yet considered by the model.

### Implications

For the time being, the model is applicable to patients hospitalized with verified Covid-19 and should support decision making on earlier discharge. Validation of the model in different regions is required to assess where it can be used in its original form and where it needs to be recalibrated. The model was developed for and its use should be restricted to this specific clinical application, if not validated for other purposes. In case the number of patients with Covid-19 in the general ward exceeds numbers that can easily be handled and thus binds resources that would be urgently needed elsewhere, the attending physicians could decide to discharge those patients with the lowest model-predicted death probabilities. It is vital that the estimated probability is not the sole criterion for decision-making and that the physician should always include a further assessment of the situation. Furthermore, it should be ascertained that discharge has no relevant impact on treatments, i.e., only those patients should be discharged where an adequate treatment can be implemented on an outpatient basis or in quarantine. It is necessary to emphasize that high estimated death probabilities should not be overinterpreted, as their reliability is not as well determined as low death probabilities, as visualized by the calibration plot. There is no general recommendation for a cut-off value, below which an earlier discharge would be justified. This cut-off depends on current strain on the healthcare system. In case patients need to be discharged, one could start with the ones with the lowest death probabilities.

## Author's Note

For commercial use of the prediction model, please contact the corresponding author and the Technology Transfer Office of the Medical University of Vienna at technologietransfer@muv.ac.at.

## Data Availability Statement

The raw data supporting the conclusions of this article will be made available by the authors, without undue reservation.

## Ethics Statement

The studies involving human participants were reviewed and approved by Ethical commission of the Medical University of Vienna (EK1315/2020). The patients/participants provided their written informed consent to participate in this study.

## Author Contributions

SHe, AZ, and AA contributed to conception and design of the study. DP, WS, KK, JS, BR, EP, MH, AS, ML, AH, KH, BL, SHe, MT, MN, CS, TS, CF, MK, GW, BJ, CT, RB-W, and HS organized the database. SHe, GS, and AG performed the statistical analysis. SHe, MF, and AA wrote the first draft of the manuscript. GS wrote sections of the manuscript. All authors contributed to manuscript revision, read, and approved the submitted version.

## Funding

This work is part of the ACOVACT study of the Medical University of Vienna and is financially supported by the Austrian Federal Ministry of Education, Science and Research, the Medical-Scientific Fund of the Mayor of Vienna (COVID024) and the Austrian Science Fund (P32064; P34783; SFB-54) and by Region Stockholm, Knut and Alice Wallenberg foundation, Jonas & Christina af Jochnick foundation (CT). The funders had no role in the design of this study.

## Conflict of Interest

The authors declare that the research was conducted in the absence of any commercial or financial relationships that could be construed as a potential conflict of interest.

## Publisher’s Note

All claims expressed in this article are solely those of the authors and do not necessarily represent those of their affiliated organizations, or those of the publisher, the editors and the reviewers. Any product that may be evaluated in this article, or claim that may be made by its manufacturer, is not guaranteed or endorsed by the publisher.
