# Supplementary material for: A Model Predicting Mortality of Hospitalized Covid-19 Patients Four Days After Admission: Development, Internal and Temporal-External Validation
Source: Front Cell Infect Microbiol. 2022 Jan 24;11:795026. doi: 10.3389/fcimb.2021.795026 (PMC8819729; doi:10.3389/fcimb.2021.795026)
Supplement: Supplementary file 1 [file DataSheet_1.docx]

**Supplemental Material**

1. **Benchmarking the Model using Machine Learning**

In order to benchmark the performance of the model competitive state-of-the-art machine learning models have been developed. For this purpose random forests (Liaw and Wiener, 2002) are trained as they showed to achieve good performance in many machine learning benchmark studies (Fernández-Delgado et al. 2014). Note that in addition random forests turned out to be less sensitive to hyperparameter tuning compared to other machine learning algorithms (Probst, Boulesteix, and Bischl. 2019, Szepannek, 2017). For this purpose forests are trained using the default parameterization ($ntree=500$ and $mtry=\sqrt{\# variables}$).

As forests consist of trees which already perform an implicit variable selection during the training process two different validation settings have been conducted: (1) forests are trained on the superset of variables including all ten blood-related predictors^[[1]](#footnote-1)^ and (2) forests trained on the subset of the eight selected variables for the final proposed logistic regression model. As model training and validation are restricted to complete cases slightly different sample sizes are resulting for (1) and (2) as they are given in table 1, together with the resulting performance of the competitive random forest models:

|  | (1) training | (1) validation | (2) training | (2) validation |
| --- | --- | --- | --- | --- |
|  | 412 | 47 | 442 | 48 |
|  | 415 | 44 | 442 | 48 |
|  | 417 | 42 | 444 | 46 |
|  | 420 | 39 | 444 | 46 |
|  | 411 | 48 | 442 | 48 |
|  | 410 | 49 | 440 | 50 |
|  | 405 | 54 | 434 | 56 |
|  | 416 | 43 | 444 | 46 |
|  | 410 | 49 | 435 | 55 |
|  | 415 | 44 | 443 | 47 |
| **AUROC** |  | **0.9236** |  | **0.9131** |

*Table 1: Resulting sample sizes for the ten training and validation folds on the different variable sets (1) and (2) as well as the mean(AUROC) of the forests on the ten validations sets.*

The results of the benchmark do not show a performance increase by using random forests instead of logistic regression and do confirm the conclusions from Bücker et al. (2021) to carefully analyze the benefits of using more complex models and to prefer simple models such as the shrinked logistic regression model otherwise. Note that both the size of the sample and the number of variables are comparatively small and thus the data are not suited for unleashing the potentials of highly flexible machine learning algorithms which in summary supports the chosen modelling approach.

1. **Validation on the B 1.1.7 Sample**

According to the shrinked logistic regression model (A) a final random forest (B) has been trained on the entire sample and the selected variable subset. Validation on the B1.1.7 sample shows a slightly better performance (AUROC = 0.8805) than the presented regression model (A) while their confidence limits (95% CI: 0.8273-0.9338, computed according to Robin et al., 2011 using 2000 bootstrap replicates) strongly overlap.

1. **Explainable Artificial Intelligence**

Modern machine learning algorithms are able to identify nonlinear dependencies between the variables without the need to specify their explicit functional type in advance as it is done in logistic regression modelling. The final random forest model (B) can thus be used to validate the functional dependencies as identified by the proposed regression model (A). For this purpose, both models (A) and (B) are compared using methodology from the field of explainable artificial intelligence (cf. e.g. Bücker et al., 2021 for an overview). In particular, partial dependence profiles (Friedman, 1997) are a popular tool to visualize how the predictions are affected by one or several predictor variables. For the purpose of our validation ALE plots (Apley and Zhu, 2020) as implemented in Biecek (2018) are used which mitigate several shortcomings of partial dependence profiles. Figure 1 compares the ALE plots for both the shrinked logistic regression model (A) as well as the random forest (B):

*Figure 1: ALE plots for all numeric variables of the entire training data of the shrinked logistic regression model (A), named baseline (blue) and the random forest model (B) in cyan.*

It can be seen that the curves of both models show the same trends. In particular, the effect of the strongly predictive variable age looks similar for both models. In contrast to the proposed regression model the ALE plots of the random forest show saturating behavior for large LDH_slopes (>50) and large Creatinine_intercept2 (>5). As it can be seen in Figure 2 these values do represent rare extreme observations. Note that Hooker and Mench (2019) state weakness of random forests to mimic the underlying data generating function in such areas of the data. In conclusion, comparison of the ALE plots confirms the predictors’ effects as they were identified by the proposed logistic regression model.

The corresponding reproducible R code of the analyses is available under https://github.com/g-rho/covid19_mortality_score.

*Figure 2: Kernel density estimation of the predictor variables Creatinine_intercept2 and LDH_slope on the training data.*

# *Apley, Daniel and* [*Jingyu Zhu*](https://rss.onlinelibrary.wiley.com/action/doSearch?ContribAuthorStored=Zhu%2C+Jingyu)*. 2020. “Visualizing the effects of predictor variables in black box supervised learning models”. JRSS A 82 (4), 1059-1086. DOI:* [*10.1111/rssb.12377*](https://doi.org/10.1111/rssb.12377)*.*

*Biecek, Przemyslaw. 2018. “DALEX: Explainers for Complex Predictive Models in R.” J. Mach. Learn. Res. 19 (84): 1–5.*

*Bücker, Michael, Gero Szepannek, Alicja Gosiewska, and Przemyslaw Biecek. 2021. “Transparency, Auditability and eXplainability of Machine Learning Models in Credit Scoring.” Journal of the Operational Research Society, DOI: 10.1080/01605682.2021.1922098.*

*Fernández-Delgado, Manuel, Eva Cernadas, Senén Barro, and Dinani Amorim. 2014. Do we need hundreds of classifiers to solve real world classification problems? J. Mach. Learn. Res. 15, 1, 3133–3181.*

*Friedman, Jerome. 2001. “Greedy Function Approximation: A Gradient Boosting Machine.” Annals of Statistics 29:1189–1232.*

*Hooker, Giles and Lukas Mentch. 2019. “Please Stop Permuting Features: An Explanation and Alternatives”. ArXiv, abs/1905.03151.*

*Liaw, Andy and Matthew Wiener. 2002. “Classification and Regression by randomForest”. R News 2 (3), 18-22.*

*Probst, Philipp, Anne-Laure Boulesteix, and Bernd Bischl. 2019. Tunability: importance of hyperparameters of machine learning algorithms. J. Mach. Learn. Res. 20, 1, 1934–1965.*

*Robin, Xavier, Natacha Turck, Alexandre Hainard, Natalia Tiberti, Frédérique Lisacek, Jean-Charles Sanchez, and Markus Müller. 2011. “PROC: An Open-Source Package for R and S+ to Analyze and Compare Roc Curves.” BMC Bioinformatics 12: 77.*

*Szepannek, Gero. 2017. “On the Practical Relevance of Modern Machine Learning Algorithms for Credit Scoring Applications.” WIAS Report Series 29: 88–96. DOI: 10.20347/wias.report.29.*

1. "PLT_intercept2", "PLT_slope", "CRP_intercept2", "CRP_slope", "Creatinine_intercept2", "Creatinine_slope", "Lymphozyten_intercept2", "Lymphozyten_slope", "LDH_intercept2", "LDH_slope", "Age", "Fever_on_admission" [↑](#footnote-ref-1)
